# Clinicians' Views on the Need for Cultural Adaptation of Intervention for Children with ADHD from the Ultraorthodox Community

**DOI:** 10.1155/2021/5564364

**Published:** 2021-05-24

**Authors:** Anat Golos, Risa Mor, Orit Fisher, Adi Finkelstein

**Affiliations:** ^1^School of Occupational Therapy, Faculty of Medicine of the Hebrew University, Jerusalem, Israel; ^2^Department of Nursing, Jerusalem College of Technology (Campus Tal), Jerusalem, Israel

## Abstract

Culture is a core context within occupational therapy, with a recent literature emphasizing the importance of cultural competence, as well as culturally sensitive assessment and intervention. The recent literature has indicated the efficacy of the Cognitive-Functional intervention (Cog-Fun) for children with ADHD among the general Israeli population, yet no studies to date have examined the necessity of cultural adaptations for minority groups. The current study examines the necessity of adapting the intervention protocol and process to the Ultraorthodox (UO) population, as perceived by occupational therapists. The study included 28 occupational therapists certified to use the Cog-Fun intervention, who reported using this approach with UO children. Participants responded to an online questionnaire developed for this study, regarding characteristics of the UO population and necessary adaptions to the Cog-Fun intervention process and protocol. Findings were analyzed using descriptive statistics and qualitative content analysis. Results of the study point to the necessity of addressing various features of the UO community, including daily routines and habits, cultural values, knowledge regarding ADHD, and accessibility of information. Participants also reported a need to adapt the graphic content of the intervention materials. The qualitative data expanded on the perceptions of the participants through four main themes: (a) knowledge regarding ADHD diagnosis and intervention; (b) perceptions and attitudes regarding ADHD diagnosis and medication; (c) factors affecting communication between the OT, parents, and teachers; and (d) adapting the intervention protocol to habits, routines, and lifestyle of UO families. This study has direct implications for therapists utilizing the Cog-Fun with UO children and may also provide insights relevant to occupational therapists using other treatment approaches with children from this culture, as well as other minority or traditional groups. Furthermore, this study may serve as an important addition to the limited literature describing cultural adaptations of evidence-based interventions.

## 1. Introduction

Culture has been defined as shared ideas, beliefs, set of concepts and meanings, values, knowledge, ways of being, customs, and often language that arises over time within a particular group [1]. Many occupational therapy (OT) models address the cultural context, which can affect a client's identity and activity choices, as well as their views regarding work, leisure, health, and self-care [2, 3]. Iwama [4] emphasized the eminent role of culture in OT, making it relevant to diverse clients' occupational needs; as such, it is considered a factor in client collaboration, models of practice, assessment tools, and therapeutic material design. In addition, recent literature addresses the need to understand both the individual client and the collective culture, as well as the interaction between the therapist and the client [5, 6].

Culturally competent practice is recognized as being necessary to meet the needs of clients, reduce health disparities among minority groups, and improve the quality of services and health outcomes [7, 8]. Cultural competency can be viewed as a contextual and dynamic process where health professionals can adjust their practice to meet the unique cultural idiosyncrasies and needs of clients with understanding and efficient communication, regardless of their client's background, ethnicity, and/or cultural nuances, and includes multiple elements such as cultural awareness, knowledge, and skills [7, 9, 10]. A synthesis of literature addressing skills for cultural competence points to the necessity of adapting practices, assessments, and interventions to the culture of individual clients [1].

In recent years, many countries are comprised of multicultural populations. Israel is an example of a culturally diverse country, which, among others, includes the Ultraorthodox (UO) Jewish population, a minority group defined by a commitment to halacha (Jewish law) [11], as well as a set of values, norms, commandments, and institutions which influence all aspects of daily living [12–14]. Aiming to preserve ancient traditions, UO are characterized by cultural conservatism and set boundaries between themselves and the general population to minimize outside influences [15]. They usually live in segregated communities and tend to have larger than average families, high fertility rates, and lower than average income [16]. The UO population is itself divided into groups and factions which differ in philosophy, expectations, and religious practice [17], a level of contact with the secular world and adherence to group norms [18].

The need for cultural adaptations and cultural sensitivity regarding treatment within the UO community has been widely recognized within different professional literatures, such as social work and health care, with adaptations to various treatment protocols being described in the literature [11, 16, 19–23]. Within the OT literature, previous studies have emphasized the need for cultural adaptation in regard to the UO population to achieve meaningful results and build an effective therapeutic relationship between the therapist and clients. Golos et al. [13] emphasize the need for cultural adaptation in OT, the importance of understanding the effects of beliefs regarding health and education, and developing culturally sensitive assessments and interventions for UO children. In addition, Engel-Yeger [24] highlights the necessity for occupational therapists (OTs) to develop competence in dealing with the various environmental backgrounds of their clients, which may involve adaptations to the assessment or intervention process, as well as setting goals which are meaningful and relevant to the client and his family. Although the need for adapted and culturally sensitive interventions has been recognized, few such adaptations have been documented in the OT literature.

Cultural factors and culturally sensitive interventions need to be considered when treating people with various health conditions, such as Attention Deficit and Hyperactivity Disorder (ADHD). ADHD is a chronic health condition within the International Classification of Functioning, Disability and Health (ICF) framework and biopsychosocial model, which affects on a medical level (body functions and structures), as well as impacts activities and social participation [25]. ADHD has been recognized as a developmental impairment in executive functions and higher cognitive processes, which play a critical role in managing the multiple and complex tasks of daily life [26]. Furthermore, ADHD has been shown to profoundly disrupt functioning in a variety of daily activities, including play, learning, self-care, and social interactions [27]. Gómez-Benito et al. [28] note the importance of considering cultural factors when working with clients with ADHD in a variety of contexts, due to the biopsychosocial nature of the health condition.

There is limited literature regarding ADHD in the UO population, but the link between religion and ADHD has been recognized [29]. Feldman [30], studying the presence of behaviors associated with ADHD among “at-risk” adolescents from the UO community, found that a large percentage presented symptoms that corresponded to DSM-IV diagnostic criteria for various types of ADHD. These findings indicate that, for these adolescents, ADHD-related behaviors contribute to their difficulty in meeting the expectations of their culture, such as regular religious studies (“Torah”) and prayer.

One OT intervention developed for children diagnosed with ADHD is the Cognitive-Functional (Cog-Fun) intervention [31], an integrative cognitive functional approach that targets cognitive, emotional, and environmental barriers to participation as they interact in an occupational context [32]. OTs must undergo a certification and training process in order to use this intervention, which is aimed at assisting children in acquiring executive strategies and self-efficacy in occupational performance. During the intervention, parents and children learn and practice executive strategies and behavioral skills together, within the context of playful activities and daily functioning [27]. Studies have indicated a positive effect of the Cog-Fun on executive functioning and quality of life, as well as a reduction of ADHD symptoms, as reported by parents, with these results being maintained in a three-month follow-up [27, 33, 34]. Moreover, a controlled study examining the efficacy of the intervention among young children with ADHD found that the positive effects of the intervention were replicable and could be attributed specifically to the intervention [34].

While the Cog-Fun intervention has been found to improve daily executive functioning among children with ADHD, no cultural adaptations for minority groups, including the UO population, have been documented. No study to date has examined the effects of the UO culture on the Cog-Fun intervention process or whether there is a need for cultural adaptation of the intervention specifically for the UO population. Therefore, the focus of the present study was to investigate whether there is a need to adapt the Cog-Fun protocol for use within the UO population, as well as which cultural adaptations are necessary to the protocol or the intervention process, as perceived by OTs. As such, the specific aims of the study were: (a) to examine the unique cultural characteristics of the UO population which may affect the intervention process; and (b) to examine the necessity of adaptations to the Cog-Fun protocol to suit the needs of the UO population.

## 2. Materials and Methods

### 2.1. Study Design

A mixed-method one-group study design was employed using both quantitative descriptive methods and qualitative content analysis of data. Mixed method research is the process of integrating quantitative and qualitative data collection and analysis to generate metainferences beyond what either approach could do alone [35]. The information was gathered through questionnaires intended to examine the necessity of adaptations to the Cog-Fun protocol for the UO population, as well as which adaptations are required, and included both scaled items and open-ended questions.

### 2.2. Participants

This study employed a convenience sampling of 28 OTs. The inclusion criteria for participation in the study are (a) certified to use the Cog-Fun intervention protocol for children (ages 5-10) and (b) experienced using the protocol with children from the UO community. All the participants were female, with over half (53.6%, *n* = 15) identifying themselves as UO, while the rest reported to be either observant (25%, *n* = 7) or secular/traditional (17.9%, *n* = 5). Further description of the study population is detailed in the quantitative results of the study (see Results).

### 2.3. Procedure

After receiving ethical approval for the study from the Ethics Committee of Faculty of Medicine, Hebrew University (No. 1507201802), a notice was e-mailed to over one-hundred Israeli OTs trained and certified to use the Cog-Fun intervention. The notice contained an explanation of the current study, as well as a link to an online survey, where participants consented to be part of the study. Participants were assured that the data collected would be used only for the current study and had the option of answering the questionnaire anonymously.

### 2.4. Measures

#### 2.4.1. Demographic and Adaptation of the Cog-Fun for the Ultraorthodox Questionnaire

For the current study, a questionnaire was developed in Hebrew by the researchers, based on the intervention protocol, and literature regarding culture, cultural competence, and cultural adaptation [7, 9, 36, 37] as well as the researcher's clinical experience and familiarity with the UO population. The researchers included experts in the fields of cultural adaptation and the Cog-Fun, as well as the first researcher who is UO. The questionnaire, which requires approximately 30 minutes to complete, examined the perceptions of the participants regarding the UO population, based on their professional and personal experience with this community. It included two main categories: demographic and professional information and questions regarding Cog-Fun intervention with the UO population, including (a) unique UO cultural factors that may impact the intervention process (e.g., knowledge and awareness regarding ADHD diagnosis, communication) and (b) specific adaptations necessary to the Cog-Fun protocol (e.g., graphic content, evaluation process). Each of the subcategories was comprised of several scaled items, rated on a scale of 1-4 according to the perceived level of relevance to the UO population (1—not at all, 2—low, 3—moderate, 4—high), as well as open-ended questions which enabled the participants to elaborate on the scaled items. Sufficient internal consistency (*α* = 0.794) was found for the questionnaires' items concerning adaptations necessary to the intervention.

### 2.5. Statistical Analysis

#### 2.5.1. Quantitative Analysis

SPSS 25 was used to analyze descriptive statistics of the demographic data and scaled items, including frequencies (means and percentages). In addition, a Kruskal-Wallis test for *a*-parametric variables was used to compare the responses of OTs who are UO, observant, and traditional/secular, in order to verify that there are no differences in responses based on the participant's religious affiliation.

#### 2.5.2. Qualitative Analysis

Replies to the open-ended questions were analyzed using qualitative approaches, which seek to arrive at an understanding of a particular phenomenon from the perspective of those experiencing it [38]. In the current study, qualitative content analysis was employed to analyze the data related to the participants' written responses. This method can be defined as a process of description of qualitative data in order to represent the clusters of responses; it involves establishing categories and identifying the frequency by which they occur [39]. The process of analyzing followed steps recommended for the technique, including preparation, organizing, and reporting of the analyzing process and the results through conceptual systems or categories. Moreover, the process of data analysis was not linear, but rather recursive with frequent reviews, in order to identify a story about the data regarding the research question [38]. Throughout the process, the key markers for quality in qualitative research, as described by Tracy [40], were addressed, including triangulation of the data by three of the researchers, one of whom identified as being UO, converging on the same conclusion, thus improving the credibility of the current study.

## 3. Results

### 3.1. Quantitative Results

#### 3.1.1. Occupational Therapists' Professional Characteristics

Of the participants, the majority had over 5 years' experience as an OT (89.3%, *n* = 25), had more than 3 years' experience using this intervention protocol (67.9%, *n* = 19), and have treated more than 10 children with this intervention (64.3%, *n* = 18). The OTs reported using the Cog-Fun protocol in various work settings, which also varied in their location throughout Israel and included cities with large UO communities (see Table 1). While all the participants reported treating children from the UO community, 82.1% (*n* = 23) also treat children from other religious affiliations, as well as UO children from various subgroups.

#### 3.1.2. Attitudes and Awareness of UO Families regarding ADHD Diagnosis and Intervention

The majority of the participants (67.8%, *n* = 19) reported that the UO population may have a moderate to high level of knowledge and awareness of the ADHD diagnosis and its effects on daily functioning, as well as moderate to high rates of compliance with pharmaceutical interventions (75%, *n* = 21). Regarding the sources from which UO families gained knowledge about ADHD, all participants believe that the source of information could be the community (including friends, family, and community institutions). Some participants also identified medical, therapy and educational staff, and printed material (e.g., books and magazines) as additional sources of information. Six participants (21.4%) reported media (e.g., internet and radio) as a source of information for UO families. When asked regarding perceptions and beliefs regarding ADHD, which are frequently found in UO communities, most of the participants (over 60%, *n* > 16) reported that UO parents believe that ADHD is a behavioral disorder, and too many children are diagnosed with it and are prescribed medications. Less than half of the participants reported that parents believe ADHD is a result of genetic and familial factors or environmental factors and can be treated with medication.

#### 3.1.3. Recommended Adaptations to the Intervention Process and Treatment Protocol

**Figure 1:**
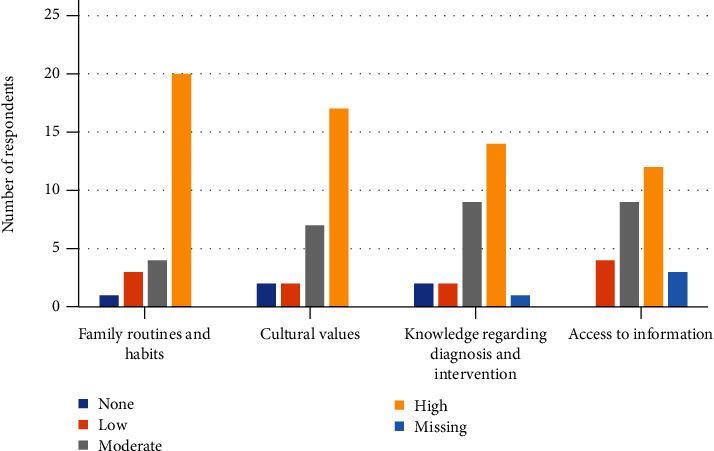
Features of the UO population that need to be addressed during the intervention process (*N* = 28).

The majority of participants (over 78.5%, *n* = 22) noted a moderate to high level of transference from the treatment setting to the home environment. However, as seen in Figure 1, they also noted a moderate-high need to address various features of the UO community during the intervention process, including daily routines and habits, cultural values, level of knowledge regarding the ADHD diagnosis, and the accessibility of information to UO families.

**Figure 2:**
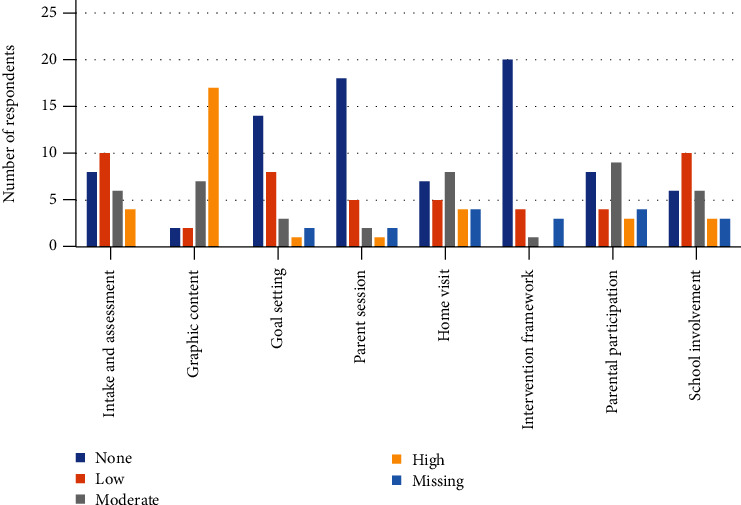
Adaptations necessary to the intervention protocol (*N* = 28).

Most of the participants (over 64%, *n* > 17) felt there was little or no need to adapt the actual Cog-Fun protocol, including initial intake and assessment, goal setting, intervention framework, and parent sessions. No conclusive need for adaptation was noted regarding home visits, school involvement, and parental participation within the treatment sessions. However, the majority (over 85%, *n* > 23) stated a moderate to high need to change the graphic content of the intervention materials (e.g., strategy symbols and the Pictorial Interview of Children's Meta-cognition and Executive Function (PIC-ME) [41] (see Figure 2).

The results of the Kruskal-Wallis test comparing the responses of participants regarding the necessity of adaptations to the Cog-Fun protocol for UO children (ten items, including the evaluation process, graphic content, and home visits), based on their religious affiliation (UO, observant, and secular/traditional), showed that there was no statistically significant difference between the groups (*χ*^2^_(2)_ = 0.28–0.02, *p* > 0.05), with a range mean rank of 9.90–17.50.

### 3.2. Qualitative Results

Qualitative content analysis of the data resulted in four main themes: (a) parental knowledge regarding ADHD diagnosis and intervention; (b) parental perceptions and attitudes regarding ADHD diagnosis and medication; (c) factors affecting communication between the OT, parents, and teachers; and (d) adapting the intervention protocol to habits, routines, and lifestyle of UO families (see Table 2).

#### 3.2.1. Parental Knowledge regarding ADHD Diagnosis and Intervention

- *Knowledge regarding ADHD diagnosis and the functional impact of ADHD*: OTs who participated in the study noted that many UO parents lacked knowledge regarding the biological basis of ADHD, as described by one participant: “[Parents] are not aware that this [ADHD] has a neurological basis- they think the child is not behaving and needs to try harder”. In addition, participants reported that while most parents had heard of ADHD as a diagnosis and related it to symptoms of excessive activity or difficulty paying attention, they often had limited knowledge regarding the functional impact of ADHD, as narrated by participants: “It is clear that most parents have basic knowledge about ADHD (mainly the symptoms like hyperactivity, impulsivity, and difficulty paying attention), but lack important information about the general difficulties these children have with executive functions and the impact these difficulties have on daily functioning.” Furthermore, participants felt that the lack of understanding regarding the biological basis and functional impact of ADHD may make it difficult for parents to accept this diagnosis for their child, particularly in the case of children who are successful in school, as described by a participant: “They [the parents] don't see these difficulties as being a result of the ADHD, they think their child is lazy, etc. Also, some children aren't hyperactive and are relatively successful academically, then the parents don't understand why he's having so much trouble, many times it's ADHD with executive functioning difficulties”
- *Parental characteristics affecting knowledge, awareness, and access to information regarding ADHD*: participants noted that different characteristics may influence parents' knowledge and awareness of ADHD, including personal and professional experience, access to sources of information (such as the internet), and information received from school or other community sources: “There is a large variance in the UO population [regarding the level of awareness]. It depends on many different factors. Families that have more than one child [diagnosed with ADHD] or mothers who work in education are sometimes more aware. There is still a lack of knowledge, especially in communities that have less access to internet Also, in my opinion, schools may give misleading information, especially boys schools.” Participants noted that occasionally, parents have inaccurate information and may be affected by stigmas that exist regarding ADHD: “The information I hear [from parents] is usually not medical or psychological, rather includes opinions and stigmas,….” Participants emphasized the necessity of addressing this lack of knowledge and reliable sources of information: “Being as parents often come with limited or disorganized information, it's very important to supply reliable and up-to-date information….” In addition, participants addressed the need to create resources which are accessible to UO families, being as a large portion refrain from using advanced media and social technology: “Since parts of the UO population have less access to technological resources (such as internet and iPhone) and are less active on social networks, it may be necessary to make reliable professional resources relating to ADHD diagnosis and intervention, more accessible to them”

#### 3.2.2. Parental Perceptions and Attitudes regarding ADHD Diagnosis and Medication

- *The tendency to hide ADHD diagnosis*: participants reported a tendency in the UO community to hide a diagnosis of ADHD from the surroundings, including the school, and sometimes, from the child himself: “There is a kind of secrecy [regarding the diagnosis], sometimes parents don't want the teachers to know about the child's difficulties and hide them from society and the child himself, then the child senses that something is wrong with him and doesn't even know what.” As expressed by another participant: “Sometimes parents refer to the medication as a ‘vitamin', and rarely- the child is not at all aware of it (for example the mother mixes it in his yogurt without the child knowing), and the parents may not be open to letting the child know he is taking ADHD medication.” According to participants, they are required to consider this tendency of secrecy during the intervention process: “In many cases, the child is not aware that he has ADHD … This needs to be considered when meeting the child and explaining the intervention and goals.”
- *Attitudes toward medication and the supportive role of the OT in finding the right medication*: the participants noted that there is a stigma in the UO population regarding the use of medications, which many times causes the parents to oppose giving it to their child: “There is a very strong stigma, almost rigidity, against the use of medication.” Some felt that it is possible to reduce the resistance to medication by clarifying the importance of medical intervention: “Most parents oppose at the beginning [to giving medication], but many change their minds after they get clear information regarding ADHD and its effects when not treated early on. Some parents will still resist.” Some of the participants noted that at times, parents had difficulty continuing to try medication, after having a negative experience (e.g., not effective or side effects) with the first prescription, but even in these cases, information and guidance by the OT are valuable: “If it's difficult to find the right dose of medication or there are a lot of side effects, many times parents will resist continuing or trying other medications. Sometimes if there is support [by an OT] at this stage, parents will continue with the follow-up”

#### 3.2.3. Factors Affecting Communication between the OT, Parents, and Teachers

- *Lack of advanced modes of technological communication*: participants noted that various factors greatly impact their communication with UO parents and affect their ability to utilize the Cog-Fun intervention protocol. One of the factors which may impede effective communication between the OT and parents from the UO population is their avoidance of advanced technology for communication, which necessitates finding alternative modes of communication, as expressed by one of the participants: “Being as most of this population doesn't have smartphones or communicate by text messaging, WhatsApp, etc. it is more difficult to communicate with them during the week, outside the appointed session, there may be a need to find creative ways to communicate, in a way that is convenient for both the parents and the therapist”
- *Accepted behavioral norms in the UO community*: participants addressed the need to be aware of the cultural differences that exist between themselves and UO parents, in order to successfully implement the intervention: “I am much more aware of the way I speak and act so I shouldn't forget that this family has specific values and it's important to respect their needs in order to build a trusting relationship. It's the same with secular families- whenever there is a cultural difference it is extremely important to improve ‘real-time' awareness because the success of the intervention largely depends on creating a therapeutic and trusting relationship between the parents and the therapist”

An example of an important cultural norm that may impact the implementation of the protocol with children from the UO community relates to the concept of modesty, which affects interactions between men and women: “…[adapting] activities, for example, being careful how I sit and which exercises or activities I do in the presence of the father, because of the concept of modesty, not to close the door, to speak less to the father than the mother. It's important to at least consider these examples and to try to sense what this family's values are and what is considered acceptable or unacceptable behavior on my part.” Some of the participants reported that these social values may cause fathers to be less involved during the intervention process: “sometimes the father won't be interested in attending the treatment or meetings as a result of issues regarding modesty, we need to be sensitive to this and allow the mother to attend on her own, and encourage her to share what is being done with the father.”

(c) *School cooperation*: participants specifically addressed the difficulty in involving and obtaining cooperation from schools: “… even when the parents want to [include the school] the teacher is not always receptive or understanding enough to act as an agent of change, it depends on the teachers' education and personal tendencies. Sometimes working with teachers can be fruitful, but sometimes there's a feeling that it's impossible to get anything moving… then you choose one main point which needs to be addressed to change perspectives and attitudes (for example: to understand what impulsivity is, or that the child isn't lazy). With female teachers, it's sometimes easier, but not necessarily.” Modesty was also addressed within the context of communication with the school: “I worked with two Hassidic children from different schools, and while one of the teachers (Rebbe) refused to speak to me on the phone and hear of adaptations that might help the child in the classroom (because I'm a woman)…”

#### 3.2.4. Adapting the Intervention Protocol to Habits, Routines, and Lifestyle of UO Families

- *Considering family routines, habits, and schedules*: the participants expressed the necessity of cultural adaptations to the Cog-Fun intervention for UO families. They noted that the daily functioning of UO families tends to be hectic and overwhelming as a result of various factors, for example, larger than average families and longer school days. As such, there is a need to understand daily routines, habits, and schedules specific to the family and adapt the intervention accordingly: “The ideal implementation [of the intervention] depends on personal adaptations: daily routines, whose home and in charge when: siblings/ which of the parents, sometimes the schedule, for example, large families tend to get through daily routines in ‘survival mode', and the OT needs to understand what is possible and what's not, something that's a burden in the short term, might not work, even if it can make things easier in the long run.” Some suggested ideas to encourage implementation of intervention plans at home: “Because the homes tend to be hectic (a large number of children, parents' work, long school days at a young age) the parents have difficulty actively transferring treatment plans. It may be necessary to consider this within the intervention framework- how to include siblings when to write notes etc. There may be a need to directly assist the parents in choosing goals together with the child which they will be able to implement within the context of their home”

When considering habits and routines in the orthodox population, it is important to be aware of, and address, gender differences, which may impact the choice of intervention goals: “UO boys don't need to prepare their schedules and some don't even bring a backpack to school every day, therefore there is no point in focusing on preparing their homework or anything to do with packing their bags or school supplies. Also, there are families where the boys are less involved at home and don't have chores, because of their long school day.”

(b) *Understanding the child's different environments*: a number of participants, especially those who are not affiliated with the UO community, described some of the strategies they use to help them bridge the cultural gaps during the intervention and to better understand the child's different environments, for example: “Many times I ask more questions to better understand situations which I am not familiar with.” In addition to culturally sensitive adaptation for the UO population in general, participants noted that different subgroups within the UO population require different adaptation, as a result of the differences between them: “The Hassidic community needs more adaptations being as they speak a different language [Yiddish] and have a different culture”; another specified: “When I treated children from Hasidic families I felt a stronger need to adapt the content and language (Yiddish), so it would be more relatable to the children (and the parents)”

One of the main environments participants felt needs to be considered is the child's home environment, particularly as the Cog-Fun protocol recommends having one session in the home to promote the transfer of treatment goals. Some of the participants expressed difficulty entering UO homes as a result of embarrassment or intimidation experienced by UO families, as noted by one of the participants: “It is much harder visiting an UO home. The concept of a home visit can be intimidating.” Another added: “… if the family is embarrassed or doesn't want people to know, their feelings need to be considered.” The difficulty entering an UO home seems to intensify when the therapist is not affiliated with the UO community, as a result of the cultural differences between the therapist and the family, as was mentioned by one participant: “Not every home is open to having someone secular visit, mainly because of the environment.” Another participant addressed this difficulty by emphasizing the need for the therapist to adapt herself to the environment of an UO home: “[the therapist needs] to respect the values of the home like dressing modestly, and asking before using a smartphone because some families are very sensitive to that.”

(c) *Addressing religious habits and values during the intervention*: participants addressed the importance of adapting the content of the intervention to the habits and religious values, which are taught and familiar to UO children from a young age, for example, checking that food meets the required level of “kashrut” (standards of dietary laws) and avoiding prohibited actions on the Shabbat: “UO children are used to inhibiting (Shabbat, milk/meat, etc.) and monitoring (e,g, checking kashrut), the more the strategies can be linked to contexts the child is familiar with the more it will be internalized.” They suggested addressing specific times during the day in which UO children with ADHD tend to have more difficulty, for example, during prayers, when children are required to sit and focus for longer periods of time: “There are differences in routines throughout the day which includes mitzvot (religious commandments). On a basic level, it is important to consider activities that are included in the daily routines, such as prayers in the morning and before bed (Shema Yisrael) which children with ADHD may have trouble with. Of course, Shabbat and holidays have social norms which need to be addressed, like sitting at the table during kiddush, etc.”

The religious habits and values of UO children have a significant impact on intervention and necessitates adapting the graphic content included in the Cog-Fun manual and materials, (i.e., the Pictorial Interview of Children's Meta-cognition and Executive Function [41]), according to norms accepted in the UO community: “I don't use the PIC-ME at all because it's not graphically appropriate- the clothing is different from those accepted in this community, etc. the goal of the interview is to connect the child to situations that arise in his day-to-day life when the child presented is very different he doesn't want to be similar to him… Also, many situations, like sitting at the Shabbat table, are not described at all.”

## 4. Discussion

The aim of this study was to examine and document necessary cultural adaptations to the Cog-Fun protocol regarding UO children with ADHD, as perceived by OTs certified to use this approach. Participants addressed various aspects of the Cog-Fun protocol and process which need to be considered when delivering service to UO families, thus supporting the literature which has emphasized both the importance of developing culturally sensitive OT interventions in general [7, 8] and interventions specific to the UO population [12, 13, 42].

Participants in the study varied in their religious affiliation, professional experience, and location within Israel, yet all reported using this approach with UO children, while many also had experience with children of other religious affiliations, enabling them to recognize adaptations specific to the UO population. It is important to note that while the participants varied in their religious affiliation, no significant differences were found between their responses based on religious affiliation.

The current study employed mixed-method research, a process of integrating quantitative and qualitative data collection and analysis, which draws on the strengths of both approaches to provide a more complete understanding than either approach standing alone [35]. The discussion integrating both qualitative and quantitative data addresses four main topics: (a) knowledge and awareness regarding ADHD diagnosis and medication; (b) the effect of cultural norms and values, on the child's routines and communication with parents and teachers; and (c) adaptations to graphic content.

### 4.1. Knowledge and Awareness regarding ADHD Diagnosis and Medication

Firstly, most of the participants emphasized the need to address the level of knowledge regarding ADHD, as well as the accessibility of reliable information for UO families. Participants reported that while parents have a basic understanding of the ADHD diagnosis, they lack clarity regarding its biological basis, as well as the effects on daily functioning. These findings are similar to those of Corcoran et al. [43] who found that parents' understanding of the biological mechanisms for ADHD was sketchy and superficial and that parents struggled with the meaning of the diagnosis.

Despite the similarities regarding the lack of clarity, a difference is noted regarding sources of information. In the current study, participants noted community sources as the most common source of information, with few responding that UO families gain knowledge from the internet or other social media. As such, while participants emphasized the importance of providing reliable information, they recommend creating resources that do not involve the internet and social media. While this is in contrast to recent literature, which has noted the rise in information-seeking online, with parents turning to the internet to fulfill their information needs, the literature does address the importance of considering barriers to internet access when using this medium to provide reliable information regarding diagnosis and intervention [44, 45].

In the current study, stigma and lack of reliable information were mentioned as barriers regarding the use of ADHD medication within the UO community. While the participants noted high rates of compliance with the use of pharmaceutical interventions, they emphasized the significant role of the OT in providing information regarding medication, as well as supporting parents through the process of trial and error. Stigma has been frequently cited as a barrier for parents to access mental health treatment, with low levels of stigma, along with parental knowledge, predicting a positive attitude regarding ADHD diagnosis and treatment [46, 47]. Corcoran et al. [43] document the ambivalence parents have regarding the decision to medicate their ADHD child and the many factors which may affect this decision, including the availability of support and information from the health care system.

The findings of the current study suggest that the difficulty in accepting an ADHD diagnosis and lack of knowledge, as well as stigma, may contribute to the tendency of UO parents to hide the diagnosis, sometimes even from the child himself. Charach and Fernandez [48] noted that while including children as active participants in decision-making may be challenging, it is a good ethical practice as well as the best way to engage them in treatment. As such, considerable studies among children and adolescents diagnosed with ADHD have highlighted the impact of their involvement and awareness on treatment-related outcomes and psychological wellbeing [48–50]. In addition, recent studies indicated that avoiding including the child in the process of ADHD diagnosis and intervention may have a long-term impact on their understanding of the diagnosis and willingness to continue treatment [50, 51].

### 4.2. The Effect of Cultural Norms and Values, on the Child's Routines and Communication with Parents and Teachers

Most of the OTs noted moderate to high transference of therapy goals to the home environment yet also reported a need to address daily routines and habits, as well as cultural values during the intervention process being as these can have an effect both in the routines of the child and the therapist's communication with the parents. The importance of cultural norms and values has been emphasized in previous literature regarding culture and cultural competence [5, 9], as well ecological theories, which contend that all activities of the family niche reflect the cultural values and other ecological variables which influence family practices [52]. Therefore, it is important to consider the lifestyle and values of a child's family unit and ethnic group as contextual and environmental factors that may affect their routines and participation [42].

When addressing routines and habits, participants noted socioeconomic factors that commonly affect the daily activities of UO families, for example, larger than average families which may impact the parent's ability to support a specific child during daily routines in order to implement and internalize content from the intervention. The importance of these socioeconomic factors was highlighted by Gilboa and Rosenblum [53], who note that OTs working with UO families should be aware that daily activities and participation patterns of many UO children may be affected by being part of a large, low-income family with young parents, as is commonly found among the UO population.

Furthermore, participants referred to the values, expectations, and religious practices that influence the daily routines of UO children with ADHD. Previous theories regarding the effect of ADHD on religious participation propose that individuals with ADHD have multiple disadvantages that affect their religious lives [29], as Feldman [30] speculated that the high rate of ADHD symptoms among at-risk adolescents in the UO community may affect their ability to participate in religious activities and societal expectations. Moreover, according to participants, the participation of UO children may be affected by gender differences in this community, which includes different academic expectations, schedules, and leisure activities. The difference between genders is noted by Engel-Yeger [42], who found differences regarding daily activity preferences between UO children and those from the general population. In addition, more gender differences were found among the UO group as compared to the secular group. The importance of considering the cultural values and norms of the family was also raised regarding conducting a home visit, as recommended in the Cog-Fun treatment protocol. Participants in the study mentioned that UO families may feel embarrassed or intimidated by this visit. Particularly, when the therapist herself is not UO, then there is an added difficulty. Participants emphasized the need for OTs to adapt themselves to the UO home environment (e.g., mode of dress, use of advanced technology) and, at times, omit the home visit. While cultural norms and habits have an evident effect on child routines and family life, the responses of the participants indicated the effect these factors have on communication with and participation of the parents and teachers in the intervention process. While there was no clear indication regarding the necessity of adaptations to these elements, participants emphasized the difficulties they experience communicating with UO parents and teachers during the intervention process.

The first barrier to communication with parents related to the technical aspects of maintaining contact throughout the week, as recommended in the Cog-Fun manual. This difficulty was attributed to the tendency of many UO families to avoid using advanced modes of communication such as e-mail, WhatsApp, and short message service. While phone calls were noted as a possible option, participants found them to be time-consuming and difficult to maintain, raising a need to develop other effective modes of communication. This barrier is especially significant, being as Cog-Fun is a family-centered intervention, with a central focus on communication with parents, which intended to enable and encourage parents to be agents of change and mediate the intervention for their child [31]. Maintaining communication with parents is explicitly recommended in order to support the implementation of the intervention in the home environment.

Aside from the technical difficulties, participants addressed the impact of social values and norms, specifically those relating to modesty, on the communication with UO fathers during the intervention process. In the UO community, modesty requires a separation between genders, including separate school systems and separation during leisure activities, as well as social and religious functions [42]. Previous literature relating to intervention with the UO community addressed the far-reaching impact of these laws as they affect the mode of dress, delivery, and behavior [12]. Participants in the current study described these practices as limiting the participation of the father in the intervention process, as fathers may be uncomfortable conversing directly with the OT or being present during certain activities. A recent literature addresses the family roles of UO fathers, in which the economic necessities require women to enter the work sphere, while encouraging men to participate in housework and be responsible for some of the child-rearing [54]. This increased involvement of UO fathers in child and domestic care increases the importance of paternal involvement during the intervention process raising a need to address culturally appropriate solutions to enable fathers to take part in the intervention.

A similar theme emerged regarding the involvement of teachers in the intervention process. While academic success and school participation are of central importance to UO parents [30, 53], participants in the current study reported inconsistent levels of cooperation from UO schools, based on personal factors of the teacher (experience, knowledge, willingness to cooperate), as well as occasionally male teachers' discomfort conversing with a female therapist.

The necessary adaptations to communication highlight the conclusion of Chiang and Carlson [55], who determined that the client's and therapist's cultures and the way their respective cultures interact are factors that affect therapy processes and outcomes [7].

### 4.3. Adaptations to Graphic Content

While the participants felt that the written Cog-Fun manual is mostly appropriate for use with the UO population, a majority expressed the need to adapt the graphic content of the Cog-Fun material such as the PIC-ME [41] and strategy symbols, as well as examples and situations presented (e.g., adapt images which include culturally unacceptable elements such as television, as well as include other common scenarios, such as participation in religious practices). Participants recommended superficial adaptations to the graphic content, as well as linking strategy acquisition to activities, habits, and values UO children are familiar with, in order to make them more meaningful to the child and family. This category of adaptation has been recognized in the literature as surface structure adaptations to evidence-based interventions, which involves changes to the original intervention's materials or activities that address observable and superficial aspects of a target population's culture, such as language, clothing, and related observable aspects [37].

### 4.4. Limitations and Directions for Future Research

While the current study adds to existing literature documenting cultural adaptations of interventions to minority groups, the study had several limitations, as well as directions for future research. First, while the study provides important information regarding the perspective of OTs, including OTs who are themselves UO, it does not address the perceptions of UO parents regarding their knowledge, awareness, and attitudes toward ADHD or the cultural fit of the intervention to their needs. To further deepen the understanding regarding this population, it would be recommended to further examine these points, using more thorough qualitative methods, such as in-depth interviews with parents, clinicians, and children, as well as other populations, who may have similar characteristics or knowledge regarding ADHD. Second, this study was aimed at gathering initial quantitative information with supporting qualitative data from a relatively small sample of female OTs, using a questionnaire that was developed for the purpose of this study. It is necessary to further study the efficacy and cultural fit of the Cog-Fun intervention with UO children, using an adapted protocol, based on the findings of the current study, then further compare the efficacy of this intervention with UO children to that of the general population.

### 4.5. Clinical Implications

This study provides guidance for OTs, who use the Cog-Fun intervention to treat UO children. In addition, the results emphasize the impact of cultural attitudes, values, and norms, on communication between OTs and clients, and may have clinical implications for therapists using other intervention models during interactions with clients from the UO population, as well as other traditional or minority groups. The results may indicate a need to expand the Cog-Fun protocol and training program, to include information and recommendations regarding adaptations relating to the specific population. Finally, this study adds to the literature describing cultural adaptations of interventions and may serve as a foundation for the adaptation of other intervention protocols to the unique cultural characteristics of the UO population, as well as an example of the process of cultural adaptation of interventions to unique cultures in general.

## 5. Conclusions

This study was the first to determine the need to adapt an intervention for ADHD children to the needs of the UO population, as perceived by OTs treating this population, using both qualitative and quantitative methods. The results of the current study indicate various cultural elements that should be addressed during the intervention process, including knowledge, attitudes, and awareness regarding the health condition, communication with caregivers, and cultural habits and values, as well as surface changes to the intervention materials. While the study provides recommendations to OTs utilizing the Cog-Fun intervention with children from the UO population, there is a need for further research to study the efficacy of an adapted intervention based on these recommendations. The findings of the study may guide therapists using other intervention models with children from the UO population. Additionally, the findings of the current study may guide therapists treating children with ADHD from other populations characterized by a lack of knowledge regarding the diagnosis, including other traditional or minority groups.

## Figures and Tables

**Table 1:** Participant professional characteristics (*N* = 28).

	*N*	%
Professional education		
BA	19	67.9%
MA	9	32.1%
Experience as an OT (years)		
0-5	3	10.7%
5-10	11	39.3%
10-15	3	10.7%
Over 15	11	39.3%
Cog-Fun work settings		
Child development center	20	71.4%
Educational setting	9	32.1%
Private clinic	12	42.9%
Work location		
Jerusalem and surrounding	20	71.4%
North	3	10.7%
Center	3	10.7%
South	1	3.6%
Missing	1	3.6%
Cog-Fun experience (years)		
1-2	8	28.6%
3-5	13	46.4%
Over 5	6	21.5%
Missing	1	3.6%
Number of children treated with Cog-Fun intervention		
Less than 10	10	35.7%
10-20	10	35.7%
20-50	6	21.4%
More than 50	2	7.2%

**Table 2:** Themes and subthemes of qualitative analysis.

Theme	Subthemes
(1) Parental knowledge regarding ADHD diagnosis and intervention	(a) Knowledge regarding ADHD diagnosis and the functional impact of ADHD
	(b) Parental characteristics affecting knowledge, awareness, and access to information regarding ADHD

(2) Parental perceptions and attitudes regarding ADHD diagnosis and medication	(a) The tendency to hide an ADHD diagnosis
	(b) Attitudes toward medication and the supportive role of the OT in finding the right medication

(3) Factors affecting communication between the OT, parents, and teachers	(a) Lack of advanced modes of technological communication
	(b) Accepted behavioral norms in the UO community
	(c) School cooperation

(4) Adapting the intervention protocol to habits, routines, and lifestyle of UO families	(a) Considering family routines, habits, and schedules
	(b) Understanding the child's different environments
	(c) Addressing religious habits and values during the intervention

## Data Availability

The data used to support the findings of this study are restricted by the Ethics Committee in order to protect participants' privacy.
